# Population Health and Paid Parental Leave: What the United States Can Learn from Two Decades of Research

**DOI:** 10.3390/healthcare4020030

**Published:** 2016-06-01

**Authors:** Adam Burtle, Stephen Bezruchka

**Affiliations:** Department of Health Services, School of Public Health, University of Washington, Seattle, WA 98195, USA; burtle@uw.edu

**Keywords:** maternity leave, paternity leave, parental leave, breastfeeding, infant mortality, low birthweight

## Abstract

Over the last two decades, numerous studies have suggested that dedicated time for parents to be with their children in the earliest months of life offers significant benefits to child health. The United States (US) is the only wealthy nation without a formalized policy guaranteeing workers paid time off when they become new parents. As individual US states consider enacting parental leave policies, there is a significant opportunity to decrease health inequities and build a healthier American population. This document is intended as a critical review of the present evidence for the association between paid parental leave and population health.

## 1. Introduction

As a society, the United States (US) spends nearly three trillion dollars each year on healthcare [1]. Debates surrounding this figure often center on methods for reducing the cost of healthcare [2], yet they rarely address why the US has such poor health outcomes when compared to other advanced countries [3]. The 1980s and 1990s saw the emergence of new hypotheses that the foundation of human health is programmed in early life [4]; there is now a substantial body of evidence supporting the importance of the period from conception through the first few years of life [3,4,5]. Still the United States remains without a strong nationwide mechanism to support this critical period, namely a law that guarantees paid parental leave. Studies that examine the effects of paid parental leave indicate that it contributes to fewer low birthweight babies [6,7], fewer infant deaths [6,8,9,10,11], higher rates of breastfeeding [12], longer parental lifespan [13] and improved mental health [14,15,16], as well as increased long-term achievement for children [17].

More than 50 years ago, President Kennedy issued an executive order creating the Presidential Commission on the Status of Women. The findings of this commission, issued in 1963, noted that the United States was lagging behind the developed world in providing support for working mothers. The Commission’s report urged: “[p]aid maternity leave or comparable insurance benefits should be provided for women workers” [18]. While maternity leave policy in the United States has progressed little in the intervening years, social norms have changed significantly. Women now make up an even greater portion of the American workforce than in the 1960s, and as a result women who become pregnant today are more than three times as likely to be working a job until the very end of their pregnancy when compared to their 1960s counterparts [19]. Of American women today who work during pregnancy, 59% will be back to work within three months of giving birth [19].

All wealthy nations other than the US have some form of legal guarantee that new parents can take paid time off to be with their child [20]. While the specifics of these guarantees vary by country, US federal legislation (the Family Medical Leave Act (FLMA)) lags behind, only provisioning *unpaid* time off for workers of companies with more than 50 employees, at which the worker must have been employed for the past 12 months and have worked at least 1250 hours in this period [6]. It has been estimated that fewer than half of American workers are eligible for this unpaid leave mandate [21]. Government officials at the highest levels, including President Obama, the US Surgeon General, and US Secretary of Labor, have all conceded that the United States continues to “stand still while family policy in the rest of the world passes us by” [20]. Although the US lacks federal provisions for paid leave, employers are free to offer paid leave to employees as they see fit [20]. While companies voluntarily offering such policies should be lauded, the Bureau of Labor Statistics has found that voluntary paid family leave policies cover only 11% of American workers [22].

Five US states have enacted laws that offer paid leave to new parents. California, while not providing job protection, does offer six weeks of leave, and 55% pay during the leave period [23]. New Jersey offers a maximum six weeks of leave, at two-thirds pay [23]. Rhode Island offers four weeks at roughly 60% pay [20]. Washington State passed a law in 2007 offering family leave at partial pay, but the law has never been funded and thus remains not in effect [24]. In March of 2016, New York became the most recent state to pass paid family leave legislation, which will phase in leave benefits over three years beginning in 2018, ultimately offering 12 weeks of leave at two-thirds pay in 2021 [25]. Additionally, Hawaii and the Commonwealth of Puerto Rico have temporary disability insurance (TDI) provisions that provide short maternity leaves (six to eight weeks) at partial wages for healing after giving birth [20].

While new parents in these regions may appreciate these policies, their statutory access to paid parental leave still falls far short of that in other developed economies. Comparing the benefits offered to a new mother in the 41 other countries considered “developed economies” by the International Labor Organization, six countries stand out as offering the shortest leave periods: Greece, Israel, the Netherlands, New Zealand, Spain, and Switzerland [26]. In all of these countries, the duration of leave available to a new mother ranges between 14 and 17 weeks, with pay at 100% for all countries other than Switzerland (which is set at 80%) [26]. California and Spain have similarly sized populations, yet a working mother in Spain can take 16 weeks of leave at full pay, while her contemporary in California can take only six weeks at 55% pay [26,27]. Japan and Canada stand out as offering the lowest rates of compensation (two-thirds pay in Japan for the first 14 weeks, with 50% pay for up to 52 weeks more; Canada offers 50 weeks at 55% pay) [26]. While both of these countries do offer fractional pay comparable to that of US states with paid family leave laws, the duration of leave offered is an order of magnitude longer.

The broader ecology of American parental leave can be seen as a gradient of benefits and access. Only 11% of American workers are privileged enough to enjoy employer-furnished paid parental leave as a fringe benefit [22], while a larger group of workers in five states has access to some partially paid parental leave. Roughly half of workers (including many of the aforementioned) have access to the 12 unpaid weeks provisioned within the FMLA [21]. At the bottom of the gradient are the remaining workers, left with no benefits at all.

Significant inequities in American health can be found along economic, educational, and racial fault lines [28]. The children most at risk of poor outcomes [29] are those whose parents are most likely to be in the 89% of workers who are not offered leave by their employers [21,22,30,31]. The top 40% of wage earners are more than 2.5 times as likely to have access to paid leave when compared to the bottom 40% [30]. The social hierarchy is itself a robust predictor of patterns of disease and mortality among populations [32], and parental leave in America traces the outlines of this social gradient. It follows that if paid leave is an intervention with health benefits, the accrual of these benefits to those nearest the top of the American social hierarchy can be seen as an inequality-generating intervention [33].

As US lawmakers at both the state and federal level consider the possibility of enacting paid family leave legislation or improving existing legislation, it will be important to consider what evidence there is for an association between improved population health outcomes and paid family leave. From country to country there is significant variation in leave policies, and the United States can leverage lessons learned from studying these policy differences. This document is intended as a review of relevant scientific literature related to paid leave and health outcomes. It is divided into the following sections: leave duration and effects on infant mortality, antenatal leave and effects on birth outcomes, breastfeeding and paid leave, and gender differences in leave-taking.

## 2. Evidence of Health Effects Related to Job-Protected Paid Leave

One aspect of the paid leave debate centers on the amount of time taken at the end stages of pregnancy and following childbirth. In 1952, the International Labor Organization, an arm of the United Nations, adopted a recommendation for 14 weeks of paid maternity leave [34]. This recommendation was updated to 18 weeks in the year 2000 [35]. Although there is variation between countries in leave length and other policy details, most developed economies offer between 18 weeks and one year of leave, at 70%–100% pay [23,26].

The earliest study we are aware of that analyzes parental leave and health outcomes was published in 1995. In this study, Winegarden and Bracy [10] used data from 17 Organization for Eonomic Cooperation and Development (OECD) countries spanning four time periods (1959, 1969, 1979, and 1989) in order to estimate the effect of maternity leave on infant mortality. Across the countries and time periods in their sample population, the average infant mortality rate was 19.4 deaths per 1000 births. Using fixed individual-effects linear modeling, they found a single week extension of maternity leave would be expected to account for a reduction of 0.5 infant deaths per 1000 births. While this is a significant finding, the authors are careful to note that this result relates to small changes around variable means and is not likely to be predictive of drastic modifications in leave benefits. This study also suffers from some methodological issues including failure to control for other variables that may be relevant, such as GDP and medical insurance coverage, and assumed linear effects of leave duration. Although these modeling issues further imply its findings cannot be widely generalized, it was a significant first study in attempts to model the health effects of leave length.

Ruhm [8], expanding on the ideas of Winegarden and Bracy, used aggregated data on 16 European countries from the period 1969–1994. Of these countries, 12 were present in the Winegarden study mentioned above. Ruhm’s analysis included many controls for confounders, including GDP, healthcare spending and insurance coverage, as well as proxies for proliferation of sophisticated medical technologies (e.g., neonatal intensive care units) and for country-specific time-varying factors that may improve child health independent of parental leave. A 10-week extension of paid leave was predicted to reduce infant mortality rates by 2.5%–3.4%. Looking more closely at infant mortality, a trend emerges where the largest improvements are predicted for later periods of infancy. Post-neonatal mortality shows the greatest effect, with a 3.7%–4.6% mortality reduction accompanying a 10-week extension of paid leave. Child mortality follows, with a 3.3%–3.5% reduction in mortality, and then neonatal mortality, perinatal mortality, and finally low birthweight (LBW).

This trend follows a certain intuition of non-linearity between paid leave and child health. Ruhm notes that in contexts where no leave is provisioned by law, such as the United States in the 1980s, most mothers will take at least one month of leave [8], so it follows that the model predicts the effects of paid leave extension will accrue after the perinatal and neonatal periods. A paid leave extension of 10 weeks would be most likely to show benefits during the post-neonatal period, and indeed that is the result of Ruhm’s analysis. Furthermore, this upward trend of effects runs counter to expected confounders, such as the dissemination of effective new neonatal medical technologies or procedures, which should have greatest effect during the first days after birth. The sharp decrease (~3.4%) in child mortality is balanced against a negligible (~0.032%) reduction in senior citizen mortality. In this case, senior citizen mortality serves as a proxy measure for unobserved influences on population health that are independent of parental leave entitlements, which again implies the validity of the model. When ‘no leave mandate’ was tested against mandates of 10, 20, 30, 40, and 50 weeks of job-protected paid leave, 40 weeks was found to have the greatest overall reduction to mortality (*p* < 0.02).

Building on the work of Ruhm, Tanaka [6] published a study in 2005 that aimed to estimate the health effects of parental leave while also including the United States in the dataset, incorporating data more recent than 1994, and including more robust controls for other health measures (such as low birthweight) and separate social policies [6]. Tanaka’s dataset spanned a period from 1969 to 2000 and analyzed the 16 European countries that were included in Ruhm’s study, plus the US and Japan. The design of his model was similar to that of Ruhm, including the same controls (GDP, healthcare insurance coverage, *etc.*), and Ruhm’s results were reproduced before the two new countries and additional six years of data were included. Tanaka found a 10-week extension of job-protected paid leave would reduce infant mortality by 2.5% overall, with analysis by period showing post-neonatal and then child mortality to have the sharpest declines (4% and 3%, *p* < 0.01) [6]. Tanaka also notes the logic of the non-linear results (that additional leave seems to accrue benefits to later child health) but points out that there is a statistically significant reduction for all infant mortality categories [6], and acknowledges that it is possible that the more leave that is available to expectant mothers, the more likely they are to use some of it before birth. One of the most important findings from both Ruhm [8] and Tanaka [6] is the substantially reduced effect for job-protected *unpaid* leave and paid leave without job-protection—it is only job-protected paid leave that seems to have the powerful effects referenced above.

This series of sequentially more comprehensive studies has shown an association between paid parental leave and lowered infant mortality, but it is important to note the authors are speaking to overall population-level trends. Although countries with more paid parental leave experienced reduced population-wide infant mortality rates even after controlling for confounders, there may also be ways in which more generous parental leave could associate with higher infant mortality risk for a given child. One possibility is that generous paid leave might entice a mother to take on employment when she would have otherwise exclusively cared for her child; Ruhm does note that his model predicts a 5.5% reduction in post-neonatal infant mortality for every 10% drop in female employment [8]. Another possibility, raised by Winegarden, is that paid leave policies may, at a population level, precipitate women having children later in life [10].

Furthermore, at least some research into infant mortality and parental leave has found mixed or negative results. An analysis that attempted to replicate the work of Ruhm and Tanaka, substituting a different dataset from 30 OECD countries, did find a reduction in infant mortality, but it was not statistically significant [36]. Dustmann and Schönberg [37], examining historical maternity leave reforms in Germany, found minimal positive effects for later reforms. They measure increased days of schooling as well as likelihood of being at a ‘high track’ school (being streamlined toward university). It is worth noting that the reforms in question added either unpaid leave (expanding from 18 to 36 months), or low-paid leave (for the period from two to 10 months, and then later 18 months). Tanaka [6] and Ruhm [8] both find that unpaid leave has very little or no effect, and other studies [38,39] often correct for partial wage replacement by using ‘full-time equivalent’ weeks or similar manipulations to correct for diminished effects. The retrospective nature of these studies, the complexity of the mathematical modeling involved, and the gaps in existing datasets all present very real challenges.

Rather than considering only paid leave extensions, another study of infant mortality examined the years surrounding the implementation of the US Family and Medical Leave Act (FMLA), which provides only for unpaid leave [40]. This study, by Rossin, found that after the FMLA implementation in 1993, benefits could be observed when looking at outcomes for groups of workers most expected to avail themselves of the new law. Less-educated and single mothers are least likely to take unpaid leave [21], and indeed Rossin’s analysis of these groups revealed no effect on the infant mortality rate of their children. When controlling for the demographic characteristics of participants who could likely afford to take the unpaid leave provided by FMLA, the analysis found a 10% reduction in infant mortality [40].

Rossin’s approach involved segmenting worker sub-populations by race, educational status, county of residence, and birth month of child, and then used difference-in-differences modeling (DD) to hold states with pre-existing unpaid leave laws as controls, while states without leave laws were taken as the treatment group [40]. Groups known to be likely to avail themselves of American parental leave laws were then compared to their reciprocals using a difference-in-difference-in-differences methodology (DDD). Rossin notes that the DDD modeling reveals a significant decrease in deaths from “ill-defined” causes (which includes Sudden Infant Death Syndrome, or SIDS) among the sub-sample (educated and/or married women in non-leave-law states), representing a 47% reduction in the likelihood that a child born to one of these women would die of an ill-defined cause in infancy.

Although Rossin’s findings may seem extreme for an unpaid leave mandate, it is important to remember that FMLA was implemented in a time when many workers did not have access to any leave whatsoever, and furthermore that context matters—extension of pre-existing paid leave in an affluent western European country with subsidized childcare, universal medical insurance coverage, and other generous social policies may have a far less pronounced effect than nationwide provisioning of leave for the first time in a setting where high-quality affordable child care, healthcare insurance coverage, and other social goods are not as evenly distributed [40].

In low and middle income country contexts, Nandi *et al.* [11] use DD modeling to predict that a one-month extension of paid parental leave will be associated with a 13% drop in infant mortality. Their study was based on more than 300,000 births in 20 countries, taking place between 2000 and 2008. Heymann *et al.* [9] also had a similar result when analyzing infant and child mortality in 141 United Nations member countries. Multivariate ordinary least squares regressions were used, with controls for per capita GDP, health expenditures, public health measures, female literacy, immunization rates, and skilled birth attendance. An increase of 10 full-time-equivalent weeks of paid leave was associated with a 10% reduction in the infant mortality rate (*p* < 0.001), and a 9% lower rate in child mortality (*p* < 0.001). Perhaps most interesting, these regressions were made for the entire dataset, as well as non-OECD countries, and the results were strikingly similar. This finding echoes that of Nandi [11], and reaffirms that paid parental leave seems to be strongly associated with lower rates of infant death regardless of whether low or middle or high income countries are analyzed. Such findings further suggest that this effect may well be causal, as the more heterogeneity that there exists between societies examined, the less likely it is that health improvements are simply due to some unobserved confounding factor(s).

A report prepared for the US Department of Labor, examining the first decade of California’s paid family leave law, is particularly instructive for shaping policy based on lessons learned. A survey of eligible workers who did not apply for benefits under the law found that one-third of eligible parents remained working because the wage replacement percentage of 55% was too low [27]. Although California’s paid leave law has increased the proportion of mothers taking leave, as well as the average amount of leave taken, multiple studies report that the average Californian mother is taking only 40% to 50% of the paid leave available to her [24,41]. This suggests that many mothers are caught at the tipping point between trying to maximize time with their newborn child and being unable to afford to take any more time off at partial-wage [27].

In 1977 Norway transitioned from three months of *unpaid* family leave to four months of job-protected paid leave, with provision for a further 12 months of unpaid leave [17]. Carneiro *et al.* [17] used Norwegian Registry data to trace the outcomes for mothers eligible for leave in 1977 (using DD modeling against matched 1975 mothers as controls). Children and mothers were followed from 1977 to 2006, providing a very broad window of observation. The study found positive effects on IQ and college attendance, with reductions in dropout rates and teenage pregnancies for children born after the addition of paid leave [17]. One key advantage of this study is that it was able to link individual children with individual mothers, rather than relying on population-level outcomes. The authors also note that the ecology of Norwegian childcare at the time of reform is important to consider, as there were no high quality childcare alternatives readily available to most parents, which differs from other studies of northern European populations. Their model included balancing of the pre-reform control to the treatment group, and additional controls for observable maternal characteristics (age, education). Controls for specific outcomes include other variables known to be positively associated with the reform to test whether the outcome of interest was due to discrete effects. Although increased breastfeeding initiation and duration is one way in which paid leave is thought to benefit child outcomes, the authors show that average breastfeeding duration did not change around the time of the reform, which they interpret to mean the observed outcome differences are attributable to more direct forms of parental investment. Perhaps most importantly, the benefits for children of mothers who were educationally disadvantaged appear to have nearly doubled. This is promising for workers in the US, where both unmarried and less educated mothers have reduced access to leave [21]. Additionally, American workers of color are twice as likely to lack access when compared to whites [42], and workers earning less than $35,000 a year are 2.4 times as likely to lack access when compared to those earning $75,000 [42]. By tracing individual registry data across the population level, Carneiro and co-authors have shown that although advantaged mothers used the newly adopted paid leave at the same rates as disadvantaged mothers, this was not the case in 1975 for their respective control groups when leave was unpaid. The implication is that access to paid leave can be a powerful intervention for reducing inequality of outcomes.

## 3. Antenatal Leave and Birth Outcomes

The US Centers for Disease Control and Prevention (CDC), in trying to illuminate inequalities in infant mortality within the United States, note that the infant mortality rate in the United States is largely a consequence of premature births regardless of race, although women of color are at higher risk for giving birth before term [29]. For babies born at 39–41 weeks, there were 2.07 deaths per 1000 births. Premature births in the 34–36 week category are subject to an average infant mortality rate more than three times as high (7.42/1000). The authors note, “Because the percentage of preterm births for all U.S. racial and ethnic groups is higher than in other developed countries, all U.S. racial and ethnic groups might benefit from prematurity prevention efforts” [29]. A different CDC publication [43] notes that 129 other countries have a lower proportion of preterm births than the US. One obvious question is whether paid antenatal leave helps prevent preterm births.

A study conducted in 1992 looked at all births (*n* = 2767) in three hospitals in Mexico City during a three-month period [44]. Occupation, working conditions, and job stress were not found to be associated with premature delivery, but lack of antenatal leave was. Also associated were maternal age, lack of prenatal care, high parity, and having no financial help in an emergency. Of all the conditions found to associate with premature delivery, no antenatal leave had the strongest correlation (a three-fold increase in risk). For those with access to antenatal leave, having used less than a month of antenatal leave carried an odds ratio of 6.3. Although this is quite high, it is important to note that it is subject to experimental confounding as some of the women in this group may have intended to use more than three weeks of antenatal leave but then went into labor prematurely. Lacking access to antenatal leave meant women in the study were over 50% more likely to have small-for-gestational-age (SGA) babies, even after adjustment for confounders. The mortality risks for SGA and premature babies are substantial, including a more than four-fold risk of infant mortality for SGA babies [45].

Rossin’s study of FMLA effects [40] found small but statistically significant reductions in preterm births for American women, as well as reductions in LBW deliveries. These results likely underestimate the potential effects of paid antenatal leave due to the FMLA being both an *unpaid* mandate and providing only a short amount of total leave (12 weeks) to be split between antenatal and postnatal periods.

Stearns, in an analysis of the effects of TDI [7], showed that even short maternity leaves can have a significant reduction in the share of low birthweight deliveries. Temporary disability insurance can be operationalized as a short maternity leave at 50%–66% wage replacement, depending on which of the five TDI states is being examined, and Stearns found the overall effect was a 3.2% reduction in LBW deliveries (*p* < 0.01). The five TDI states (CA, HI, RI, NJ, NY) were examined individually and as a group using a DD model balanced against synthetic control states. Synthetic controls were constructed using a weighted average of non-TDI populations, with matching for statewide percentage of LBW, maternal age, marriage prevalence, and race. Because not all pregnant women in TDI states during the sample period took leave under TDI, the overall stated effect is an underestimate. California’s historical data on disability claims reveals that 27% of women who gave birth in that state during the study period (1979–1985) made a pregnancy-related TDI claim. A treatment-on-the-treated (TOT) effect can therefore be calculated by dividing the overall decline in CA LBW (3.2%) by the proportion of eligible women who did take TDI (0.27), for a treatment effect of 12% reduction in LBW. This is quite significant, considering that TDI leave length is just a few weeks and therefore most women are likely to use only a portion (if any) of it for antenatal leave. Stearns finds TDI reduced “early term” births (37–38 weeks gestation) by 6.6%, but had almost no effect on premature (before 37 weeks gestation) births. The increased number of full-term births due to this reduction accounts for roughly 40% of the observed LBW reduction.

An analysis by Del Bono and colleagues [46], using data from the CDC’s National Survey of Family Growth, found smoking during pregnancy increased the risk of low birthweight by 5%. Early work stoppage is associated with significant improvements for birthweight and preterm delivery, independent of smoking. Additionally, Del Bono *et al.* estimate that the effect sizes of stopping work early are one-half that of smoking during pregnancy in terms of absolute value. A study in Canada of 363 recent births to working mothers found that the increase in duration of antenatal leave was significantly associated with reductions in birth complications [47]. This is corroborated by a nested case control study of 447 working women in California which revealed that those taking antenatal leave during or after the 36th week of pregnancy experience nearly four times lower odds of cesarean delivery (OR: 0.27) [48].

Currie and Rossin-Slater [49] showed that having experienced a hurricane during pregnancy increases the probability of labor and delivery complications. They suggest that the effects of this stress may be too subtle to observe through more common markers such as gestation length and birthweight and that further research is needed to better understand the effects of stress during pregnancy.

Preterm delivery and low birthweight are related outcomes, with evidence that third trimester stress may be a significant driver of preterm delivery [5,50]. Present knowledge of human biology as well as animal models both support the notion that stress during pregnancy can create lasting harm to offspring [5,51]. Paid antenatal leave offers promise as a population-level intervention for improving birth outcomes.

## 4. Breastfeeding, Immunizations, and Maternal Health

There is also evidence that parental leave can affect breastfeeding behavior. Baker and Milligan [12] found that following a six-month extension of Canada’s paid parental leave, there was a 40% increase in exclusive breastfeeding at six months, and an average three-to-3.5-month increase in paid leave utilization. When examining health outcomes reported by parents to Canada’s National Longitudinal Study of Children and Youth (NLSCY), the authors could not find any long-term benefit to child or maternal health captured by the data in the NLSCY survey, despite having chosen outcomes purported to be linked to breastfeeding. In addition to variables related to breastfeeding and child health (including asthma, allergies, and ear infections), self-reported maternal health and maternal depression were also included. The authors report that the maternity leave extension (and attendant increase in breastfeeding) did not have a consistent, robust effect on child health as captured by NLSCY data. It may be that the NLSCY survey is not sensitive enough or otherwise does not capture the benefits of breastfeeding increase. Still, several breastfeeding studies have reported associations between breastfeeding and these indicators [12], so Baker and Milligan’s findings illustrate the possibility that prior studies may overestimate certain effects, or the effects may depend on context. Khanam *et al.* [52], examining asthma and bronchiolitis in children of Australians found statistically significant reductions for paid maternity leave, but the relationship was no longer significant if parental health was included as a covariate. This might imply parental leave acts as a safety net for those parents whose own health is compromised. Perplexingly, paid paternity leave was associated with a rise in asthma, although this may be a case of reverse causality (fathers with asthmatic children are more likely to take paid leave).

In the US, two groups of breastfeeding data from the Infant Feeding Practices Study were analyzed (one group before California’s paid family leave law implementation, one after) and researchers found evidence of improvement in breastfeeding practices after the law was implemented: a 3%–5% increase for exclusive breastfeeding at three and six months, and a 10%–20% increase for ‘some breastfeeding’ at the three-, six-, and nine-month marks [53]. The authors of this study are careful to note that as with many natural experiment studies, there are many unseen confounders for which they may not have adequately controlled. Another study that attempted to answer this question in the United States looked at breastfeeding practices among more than a thousand American mothers [54]. Returning to work within 12 weeks, and returning to work after 12 weeks while working more than 34 h per week were both associated with significantly shorter breastfeeding duration.

According to the CDC, only one in five American mothers exclusively breastfeed for the recommended six months [55]. Although there are both physiological and cultural reasons why breastfeeding initiation and continuation can be difficult for new mothers, a survey of UK mothers found 90% of those who give up breastfeeding within six weeks of birth report they would have liked to have breastfed longer [56]. American mothers who plan to return to work within six weeks are 40% less likely to breastfeed exclusively when compared to those planning to take 12 or more weeks of leave [57]. Whether leave from work lasts a few weeks or a few months, approaching the end of leave often creates major problems for breastfeeding. Returning to work emerges as the top reported reason for cessation of breastfeeding after the first two months [56,58]. One 2010 study that followed more than 20,000 mothers in Taiwan found that maternal return to work within six months was a significant barrier to breastfeeding initiation and continuation [59]. Taiwan’s statutory maternity leave is eight weeks in length, and at the eight-week mark the prevalence of breastfeeding was more than twice as high for mothers who did not return to work as compared to those who did [59]. In America, mothers taking maternity leave experience a four-fold increased odds of failure to establish breastfeeding for those who return to work in fewer than six weeks, and a two-fold higher odds for those who return during the six- to 12-week period, relative to women not returning to work, after adjusting for covariates [48]. Another study using propensity score modeling (which has the effect of not needing to adjust for covariates as both the treatment and comparison group are matched) found a less drastic but still statistically significant effect [60].

A multitude of studies and several systematic reviews have shown breastfeeding to associate with a lower risk of SIDS [61,62,63,64], necrotizing enterocolitis [62,63], lower respiratory infections [61,62,63,65], high blood pressure [66,67], diabetes [62,63,66,67], asthma [61,62,63], obesity [62,63,67], and childhood leukemia [61,62,63]. Breastfeeding is also positively associated with childhood cognitive development [66,68,69], although the evidence here is less clear. Recent criticisms have been raised with respect to the quality and design of many breastfeeding studies. Some study designs do not adequately distinguish between “any” and “exclusive” breastfeeding practices [62]. Essentially all studies are captive to ethical issues that prevent randomized interventions, and observational studies may overlook important outcome determinants [12]. So, while it is important to note that the CDC [55], US Surgeon General [70], American Academy of Pediatrics [62], and World Health Organization [53] endorse six months of exclusive breastfeeding for infant health, there is some controversy as to whether observational research may overestimate breastfeeding benefits. Several of the reviews cited above make a distinction between strongly supported evidence and that for which there is mixed support; SIDS accounts for 21% of American infant mortality [62], and is strongly linked inversely to breastfeeding [61,62,63,64].

Increasing prevalence of childhood immunizations is one indirect way in which parental leave might improve health. The evidence is mixed, with Tanaka [6] finding no change in immunization prevalence among his study countries, yet other analyses have found increases in both the US [60,71] and on a global scale [38,72]. Khanam *et al.* [52], found that the probability of being fully immunized fell by more than 20% for Australian children whose parents had no access to any parental leave (in this case, while paid leave was more effective, any leave at all appeared to be the determining factor). Differences in culture and public health policy between European countries (which comprised the bulk of Tanaka’s study) and Australia may help explain the disparity in findings between different investigations. In the case of infant mortality, the beneficial relationship between child death and paid parental leave appears quite robust. There are a multitude of pathways through which paid leave could help to prevent infant mortality. Whether boosting rates of vaccination against diseases such as measles and pertussis is one such way may depend on social norms and public health policy within each nation.

Whether paid leave improves maternal health is another important consideration, and a recent systematic review of maternal health outcomes found mixed results [14]. At a policy level, studies included in the review found either no effect or a negative association. Studies conducted at an individual level all reported associations with better outcomes for indicators including physical health, intimate partner violence, and psychological distress. Other individual-level studies of maternal depression have also reported positive associations [15,16]. Again, the trend seems to be that there are substantial challenges in undertaking observational studies, and findings depend on the manner in which the problem is operationalized as well as the methods of capturing the potential effects of a policy or exposure.

## 5. Gender Issues

Childcare and parental roles are often highly gendered in Western societies. In even the most progressive societies, with the most generous paternity and parental leave provisions, women still take substantially more child-related leave than men [26]. Sweden became the first nation to offer paid paternity leave in 1974 [73], with 39 other nations implementing such policies by 1994, and a total of 79 countries by 2013 [26]. Paternity leave in some nations is a fairly new concept, while in others it is now decades old. Embedded within the structure of the paid leave laws are several distinctions that can have important ramifications to either magnify or reduce gender inequity.

Studies of leave policies that allocate sizeable non-transferable portions to the father have revealed higher leave enrollment by fathers [39,74,75]. Additional leave, usually available as shared leave that can be taken by either parent, is used nearly exclusively by the mother [26]. In Israel, less than 1% of fathers take paternity leave [76,77]. This contrasts with Iceland, where more than 80% of fathers take leave, with an average of 99 days, compared to 178 for mothers [26,77].

Examining the paid leave structures of both Iceland and Israel, it is apparent that the nuances of paid leave policy are a determinant of gender imbalances in leave-taking. In Israel mothers get 14 weeks of paid maternity leave, and there is no statutory provision for paternity leave (defined here as leave reserved exclusively for the father) [26]. Rather, the father may take leave with the consent of the mother, by taking a minimum of three [26] (and a maximum of five) [76] weeks of her leave. Fathers can only do this if the mother consents and is also qualified for maternity leave (meaning working fathers with unemployed wives will not be eligible [78]). The result is that very few Israeli fathers take paternity leave. In Iceland, where three months are reserved for mothers, three for fathers, and three to be shared as desired, many fathers take leave [26]. Iceland achieved a doubling of the average number of days taken by fathers in just a seven-year period thanks to an extension of father-only leave. Norway and Sweden have seen similar improvements by implementing periods of exclusive leave, shifting from fewer than 5% of fathers taking leave to ~90% [73,74]. By shifting from *unpaid* to paid leave, California managed to double the proportion of fathers taking parental leave [20].

Interestingly, many countries with a sharply gendered imbalance in leave-taking have managed to make striking and rapid shifts toward equality by making modest changes to their parental leave structure [79]. The message is that various means of incentivization can help to make leave-taking less gendered. Wage replacement (in high proportions), job protection, offering sufficient shared leaved for both parents, and reserving leave exclusively for fathers (leave that will otherwise be ‘lost’ if not taken) all appear to strongly influence take-up rates [26,79]. The hope for many is that this shift may not simply reflect a different number of leave days taken, but also catalyze an appreciation for the contributions that each partner makes to the partnership and to the well-being of the child [79]. Investigations into the patterns and behavior surrounding parental leave have suggested that paternity leave reforms have the ability to change the social meaning of fatherhood [80]. Paid paternity leave reforms may be a way to strengthen the family unit, ease the burdens of maternity, and, in normalizing male parental leave, reduce the implicit workplace discrimination against women.

Studies on paternity leave often investigate marital roles, and households where the two parents share a more even distribution of leave have been found to have more cooperation between parents [79]. The longer the period of leave offered to fathers, the more they are involved with their infants and families [71,81,82,83,84,85]. Paternal involvement has been significantly associated with emotional, psychological, behavioral, and cognitive benefits to children [71,85]. Paternity leave also has a positive effect on the return of mothers to the workplace [86] and has been associated with increased rates of breastfeeding [87].

While nearly every study that seeks to measure paternity leave effects ranges from finding no result to finding positive outcomes, at least one study, by Norstrom *et al.* [88], finds some negative outcomes when fathers take significant amounts of paternity leave. Examining a subset of the Swedish parent/child cohort of 1988 and 1989 (*n* = 105,786), they found couples taking a more gender-equal amount of leave had children who were more likely to be treated for depression and anxiety. Depression and anxiety were detected by ICD-10 code usage related to outpatient medical visits and prescription of medication, from records obtained via the Swedish Outpatient Register and Swedish Drug Register. Gender-equal leave was defined as each partner taking at least 40% of the total leave. The implication is that there may be some negative effects of more equal co-parenting. One alternate explanation is that a mindset that leads to more gender-equal parenting is also predictive of parents that are more receptive to symptoms of depression or anxiety (*i.e.*, a surveillance bias), leading to higher rates of *diagnosis* among children in these families, and that treatment may not be a perfect surrogate marker for background prevalence.

Investigating the possible health benefits of paternity leave, Månsdotter and colleagues [13,73] have twice examined the Swedish Multigenerational Register for men who became fathers in Sweden in the late 1970s and 1980s, and found significantly reduced odds of early mortality among fathers who took paternity leave. Dividing the fathers into groups based on the amount of leave taken, they found the protective pattern followed a dose-response relationship for leave lengths up to a few months. This pattern persisted even after adjustment for age, income, education, and country of birth. These studies have not been replicated elsewhere, and should be viewed with attendant caution, but raise an interesting question that is worth further exploration.

Owing to the fact that taking significant paternity leave is not necessarily a normative behavior, even in countries that may extend this option, it is possible there are unobserved characteristics related to fathers who take leave, and these characteristics are confounding some study designs. Some investigators speculate about the potentially toxic nature of masculinity (e.g., a propensity for more health-destructive behaviors such as alcohol use) and the notion that childcare stands in opposition to masculinity, possibly introducing a bias where men who self-select for parental leave exhibit healthier behaviors [13]. Analyzing masculinity and paternity leave has shown that while masculinity is associated with all-cause mortality, and paternity-leave-taking also has a relationship to all-cause mortality, there is no association between masculinity (as measured) and paternity leave [73]. The data from some studies of childcare by fathers suggest that the quality of the time invested is more critical than the overall quantity [85], which may make paternity leave outcomes difficult to analyze quantitatively. More research is needed to improve our understanding of the possible intersectionality between fatherhood, paternity leave, masculinity, child health, and parental health.

## 6. Conclusions

Of working US women pregnant with their first child, 87% report [19] working through the last month of pregnancy—is this a choice made by women who love their jobs, or an unfortunate reality for expectant mothers who have no other option? Millions of American women each year face the challenges of getting time away from work to attend prenatal checkups, to bond with their newborn, to initiate and continue breastfeeding for several months, to ensure their child is fully immunized, and to do all of this in the face of financial adversity and a fear of being fired for taking too much time off. The overall trend in research on the health consequences of parental leave is that parental leave supports two precursors to improved child health—breastfeeding and immunizations—and potentially reduces maternal stress and depression. In light of the challenges that new parents face, it is perhaps not surprising that a plurality of studies find that access to paid parental leave strongly associates with lower rates of mortality throughout infancy and childhood.

The United States has an infant mortality rate at least twice that of Sweden [89]. About 39% of US excess infant mortality when compared to Sweden is due to our high preterm infant mortality rate [89]. Lacking antenatal leave has been associated with a three-fold increase in risk [44] of preterm delivery. Working longer into pregnancy has also been linked to delivery complications [47,48]. Taking leave before the end of pregnancy has benefits to birthweight approaching the same magnitude as the harms seen in smoking during pregnancy [46,51]. Examining 18 countries over more than 30 years, Tanaka [6] found a statistically significant correlation between lower birthweight and lack of access to job-protected paid parental leave. Stearns found the treatment effects for even short maternity leaves meant a 12% reduction in LBW deliveries to mothers in the five American states with TDI programs [7]. Rossin [40], examining the effects of unpaid leave for women believed most likely to take such leave, found a 47% reduction in the likelihood an infant born to one of these women died of an ‘ill-defined’ cause. SIDS, one of several causes of infant mortality in the ‘ill-defined’ category, makes up 21% of American infant mortality alone [62].

The complex and multifactorial nature of infant mortality itself, which is a term encompassing many disparate causes of death, presents serious challenges for investigation. Further complicating matters, paid leave may be protective against infant mortality through many pathways, not all of which are immediately obvious. We do know that paid leave for mothers is linked to increases in breastfeeding rates [12,53]; we also know that returning to work when leave benefits are exhausted is often cited as the top reason for breastfeeding cessation [56,57,58]. Mothers in America who take short leaves face double or even quadruple the odds of failing to initiate breastfeeding when compared to those who do not work [54]. The American Academy of Pediatrics says infants not breastfed face more than 3.5 times the odds of SIDS mortality when compared to exclusively breastfed babies [62].

Several credible examinations of paid parental leave in dozens of countries, from low and middle income to wealthy ones, have demonstrated a strong link between paid parental leave and child survival. Investigations by Ruhm [8] and Tanaka [6] have shown a non-linear effect on the health benefits of parental leave, with the greatest improvements seen after children are a month old. The more parental leave is extended, the greater these benefits appear to be (at least up to the 40-week mark). Ruhm estimates implementing 40 weeks of job-protected paid parental leave to accompany a 15% reduction in child mortality—a reduction that would correspond to more than 4000 fewer child deaths each year in the US [89].

The United States has a policy of just 12 weeks of unpaid parental leave, a policy that covers only half of its workers [21]. In the few states that mandate paid parental leave, the fractional wage replacement is low, the length of leave is only a handful of weeks, and there may not be job protection. These policies serve to further entrench existing health inequities. The children most at risk of not being breastfed, most at risk of being born preterm or low birthweight, most at risk of dying in the first years of life—these are the children of parents most at risk of not being able to take leave from work. The lack of policies substantially benefitting early life in the United States constitutes a grave social injustice: those who are already most disadvantaged in our society bear the greatest burden.

The experiences of other nations can give us hope. When Norway converted from 12 weeks of unpaid leave to 18 weeks of paid job-protected leave, a range of benefits in child achievement could be observed across the following decades [17]. Most importantly, although these improvements could be seen across the population, the benefit to children of disadvantaged mothers was roughly doubled. The implication of this research is that paid parental leave may be an intervention for not just improving child health, but for reducing inequities in health and achievement.

Outcomes such as infant death may occur through a number of pathways, and when an intervention has subtle effects on multiple determinants of an outcome—decreasing stress during pregnancy, allowing more time for prenatal checkups, improving birthweight and decreasing rates of preterm delivery, increasing the likelihood and duration of breastfeeding, increasing the probability of a child being fully vaccinated, allowing more time for bonding and infant care—it seems to be the cumulative effect of these (and other yet unobserved) pathways that accounts for the association with higher rates of infant and child survival. If the benefits of paid parental leave on child health are manifold, it follows that attempting to isolate and quantify the effects of any one potential benefit will likely present a significant challenge. When researchers examine discrete pathways, for example vaccination prevalence, the findings can vary drastically based on context. That some pathways may be more important than others depending on context can help to explain why lower infant mortality rates are associated with longer-paid, job-protected parental leave in a variety of settings.

Accounting for the qualitative nature of these topics is important for understanding variation in outcomes, and as scientists we must work to go beyond simply quantifying time spent, or lives lost, or likelihood of improvement. We must attempt to capture the nature of experience and agency, and to understand how experience begets biology. More research is required to better elucidate the links between human health and paid parental leave. However, given the body of existing evidence, parental leave policies that support all working families by providing parents the opportunity to be with their newborn for an appropriate length of time should be considered as a population health priority in the United States.
